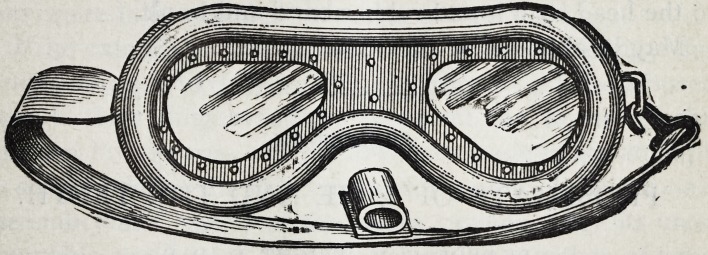# A New Invention Useful to Dentists Working Continuous Gum and Plate Work

**Published:** 1884-05

**Authors:** 

**Affiliations:** Clinical Instructor Dental Department University of Maryland.


ARTICLE II.

### A NEW INVENTION USEFUL TO DENTIST'S WORKING CONTINUOUS GUM AND PLATE WORK.

INVENTED BY DR. GENESE, CLINICAL INSTRUCTOR DENTAL DEPARTMENT UNIVERSITY OF MARYLAND.

It consists of an eye-guard which can be made air-tight for those working in the Chemical Laboratory, and ventilating for working at the gum furnace, and plate work, platina melting and assaying. In the first-mentioned work it is impossible for fumes of acids, smoke or dust to reach the eyes, and when using it with the ventilating tubes open, it is still air-tight around the orbital surface, while the air has to pass some distance through tubes, making it impossible for the eyes to get injured by the glare of flame and heat of the glowing charcoal while soldering and the gas emitted therefrom which is so injurious to the eyes.

It is a non-conductor of heat, and therefore valuable at the furnace to enable the dentist to examine the work in the muffle closer and without discomfort.

It is light and comfortable, and does not distress the wearer like ordinary goggles or glasses hitherto used for that purpose of protection.

Mica being used for the lenses, gives a softer light without altering the focus of sight like glasses, no condensation takes place. Breaking is impossible from change of temperature, and they can be bent in any form without injury.

The ventilating is accomplished through the tubes forming the fastenings, which are composed of a hollow tube bent and soldered to the frame, the distal end is pierced with fine holes to pass air and can be made air-tight by plugging the inner hole which passes through the frame to the inside.

The wood cut shows the entire instrument and a section of the rubber tube.

The frame is composed of pure lead stamped to an inner and outer frame, with edge up.

The mica is the size of the inner frame, and is secured by a double layer of rubber cement riveted and then hermetically sealed by soldering the frames at the edges by this process, the frames forms a box in which to lay the pad, which is a tube of rubber inflated and made in one piece on a flat band (and vulcanized in steel moulds.) This band and tube is again cemented to the frame and the outer and inner edge milled over the band projecting from the tube of which a section is shown in the engraving. It is adjusted to the head by a broad rubber band and hook.

May be obtained at all Dental Depots.

## Figures and Tables

**Figure f1:**